# Assessment of Renal Osteodystrophy via Computational Analysis of Label-free Raman Detection of Multiple Biomarkers

**DOI:** 10.3390/diagnostics10020079

**Published:** 2020-01-31

**Authors:** Marian Manciu, Mario Cardenas, Kevin E. Bennet, Avudaiappan Maran, Michael J. Yaszemski, Theresa A. Maldonado, Diana Magiricu, Felicia S. Manciu

**Affiliations:** 1 Department of Physics, University of Texas at El Paso, El Paso, TX 79968, USA; mcardenas9@miners.utep.edu; 2 Border Biomedical Research Center, University of Texas at El Paso, El Paso, TX 79968, USA; 3 Division of Engineering and Department of Neurologic Surgery, Mayo Clinic, Rochester, MN 55905, USA; Bennet.Kevin@mayo.edu; 4 Department of Orthopedic Surgery and Biomaterials and Histomorphometry Core Laboratory, Mayo Clinic, Rochester, MN 55905, USA; Maran.Avudai@mayo.edu (A.M.); Yaszemski.Michael@mayo.edu (M.J.Y.); 5 Department of Electrical and Computer Engineering, University of Texas at El Paso, El Paso, TX 79968, USA; tamaldonado@utep.edu; 6 Touro College of Osteopathic Medicine, New York, NY 10027, USA; dmagiric@student.touro.edu

**Keywords:** renal osteodystrophy, statistical analysis, Raman spectroscopy, label-free detection, multiple biomarkers, diagnostic devices, artificial intelligence

## Abstract

Accurate clinical evaluation of renal osteodystrophy (ROD) is currently accomplished using invasive in vivo transiliac bone biopsy, followed by in vitro histomorphometry. In this study, we demonstrate that an alternative method for ROD assessment is through a fast, label-free Raman recording of multiple biomarkers combined with computational analysis for predicting the minimally required number of spectra for sample classification at defined accuracies. Four clinically relevant biomarkers: the mineral-to-matrix ratio, the carbonate-to-matrix ratio, phenylalanine, and calcium contents were experimentally determined and simultaneously considered as input to a linear discriminant analysis (LDA). Additionally, sample evaluation was performed with a linear support vector machine (LSVM) algorithm, with a 300 variable input. The computed probabilities based on a single spectrum were only marginally different (~80% from LDA and ~87% from LSVM), both providing an unacceptable classification power for a correct sample assignment. However, the Type I and Type II assignment errors confirm that a relatively small number of independent spectra (7 spectra for Type I and 5 spectra for Type II) is necessary for a *p* < 0.05 error probability. This low number of spectra supports the practicality of future in vivo Raman translation for a fast and accurate ROD detection in clinical settings.

## 1. Introduction

Bone is a dynamic tissue model. Consequently, a constant remodelling process, which is known as bone turnover, occurs throughout the life [1]. During this metabolic process, a variety of molecules are released into the circulatory system and have been identified as bone turnover markers (BTM) [1,2,3]. Renal osteodystrophy (ROD) is an exclusive diagnosis of bone abnormal mineralization and morphological changes in strict relationship with skeletal chronic kidney disease-mineral and bone disorder (CKD-MBD) [4]. As part of bone quality, which is a commonly used terminology to describe bone health, ROD manifests itself with abnormality in bone turnover rate [4,5,6]. Evaluation of bone quality is usually derived from both biological and clinical perspectives, and encompasses all presently known bone abnormalities in the following categories: bone turnover, mineralization, bone volume, linear growth, strength, as well as soft tissue and vascular calcifications [4,6,7,8,9,10]. As a descriptor of the bone turnover rate, BTM varies over a significant range. In low-rate turnover, cancellous bone volume and trabecular bone thickness are lower than those of normal-rate turnover, whereas in high-rate turnover an opposite trend is present, with increases in volume and thickness [6]. Concerning BTM ratios, high-rate turnover exhibits less minerals within the tissue, leading to reduced mineral-to-matrix ratios. Lower carbonate-to-phosphate ratios are also encountered in comparison with those for low-rate turnover [6]. Despite variances in bone turnover occurring in ROD, the overall bone quality is the primary and major indicator to determine if ROD is present.

Since bone metabolism is complex, detrimental changes within the bone structure may develop during early onset of ROD, and become more acute with continuous degradation of the kidney function. As the kidney fails, there is a progressive disruption of mineral and waste homeostasis within the body. The main effect is the kidney’s influence on circulatory levels of phosphate and calcium. With the deterioration of the kidney’s ability to process these analytes, increased concentration of serum phosphate is observed. Excess serum phosphate binds with available calcium, and being a non-homeostatic event, the serum concentration of calcium decreases. In response, the parathyroid glands release parathyroid hormones (PTH) to increase serum calcium levels towards normal homeostasis levels. In CKD, the kidney cannot respond to PTH correctly, due to impairment of the kidney’s ability in converting 25-hydroxyvitamin D (also known as the pre-hormone calcifediol) to the hormonally active metabolite 1,25-dihydroxycholecalciferol (known as calcitriol). Calcitriol serves as the primary response to PTH, by increasing serum calcium levels through stimulating calcium uptake from the intestines. However, in kidney dysfunction, the synthesis of calcitriol is severely impacted, leading to a disruption of the calcium uptake from the gut. Consequently, the skeletal system being a large calcium reservoir is targeted, serving as the initiation of ROD. Thus, individuals with advanced CKD tend to exhibit more severe forms of ROD and extensive incidence of bone fractures [6,7,8,9,10]. It has been also reported that ROD can be a sign of metabolic aging [11,12].

Noninvasively, bone assessment is usually performed by dual energy X-ray absorptiometry (DEXA) or quantitative computerized tomography (QCT)-based methods [7]. While DEXA and QCT image the areal bone mass and volumetric bone mineral densities, respectively, they are still lacking the necessary resolution for detection of bone architecture disruption [13,14]. Thus, accurate clinical evaluations of bone remodelling and of ROD diagnosis are still accomplished using invasive transiliac bone biopsy followed by in vitro histomorphometry [7]. However, even if histomorphometry remains the gold standard technique, it lacks potentially in vivo translation. The main reason behind this failback is the compulsory sample staining in histomorphometric analysis. Furthermore, assessment of disease progression and its response to different treatments requires for additional invasive biopsies, since each histomorphometric investigation only provides data on a single point in time, preventing the time correlation of different histomorphometric patterns with that of fracture risks. This situation emphasizes the need for improvement in analytic techniques for minimally to non-invasive alternative analysis that could provide similar or better degrees of diagnostic information. Specifically, the need for techniques that could provide in-depth information regarding bone’s quality, progression of ROD by observing BTM, and risk evaluation of bone fractures.

In comparison to the clinically implemented QCT and magnetic resonance imaging (MRI) procedures and related high cost (they can be performed mainly in well-equipped facilities affiliated to hospitals), besides histologic techniques, spectroscopic methods of investigation such as Raman and Fourier transform infrared (FT-IR) spectroscopies are more commonly used in research laboratories. Not only are they more available, but also they allow for a more detailed examination of bone quality parameters at once, without the requirement of histological sample staining. Whereas both spectroscopic techniques provide a comprehensive chemical analysis that is useful for understanding likelihood of fractures and is complementary to that from QCT and MRI, relating to potentially in vivo translation, Raman spectroscopy is superior due to its insensitivity to water absorption. However, despites their accessibility, there are only relatively few reports on such Raman studies [15,16,17,18,19,20,21,22,23,24,25], with a slightly larger number of FT-IR investigations [26,27,28,29,30].

In a recent Raman spectroscopic study, we demonstrated that the bone samples of patients with ROD exhibit an overall increase in phenylalanine and decreases in calcium content, in mineral to matrix ratio, and in carbonate to matrix ratio [25]. Since just a single Raman spectrum is clearly not sufficient to assess at statistically significant levels the differences between normal and ROD samples, we took advantage of confocal Raman microscopy. Thus, by accumulation of a large number of independent spectra (22,500 spectra for each Raman mapping), identification of the samples with an excellent accuracy (less than 10^−300^) was achieved. All these significant biomarkers (i.e., phenylalanine, phosphate, carbonate, amide content, mineral-to-matrix ratio, and carbonate-to-matrix ratio) were simultaneously determined with this unprecedented accuracy. The power analysis showed that for each biomarker, a relatively low number of spectra (of the order of 20–50 spectra) was required to identify the ROD samples at the typically desired level of significance (*p* = 0.05).

The current research, while being a logical continuation of our previous efforts, also seeks to advance this work by simultaneously considering all of these biomarkers in answering the question concerning the minimum number of spectra required to accurately classify an unknown sample. By utilizing artificial intelligence approaches and advanced statistical analysis of data, we attempt to prove the viability of in vivo Raman translation based on future development of an optical-fiber-based biosensor to allow data collection and signal multiplexing through a partially invasive needle biopsy procedure.

## 2. Materials and Methods

### 2.1. Sample Preparation

The samples analyzed in this work were received from the Mayo Clinic, in Rochester, Minnesota, and consist of 7 iliac crest bone specimens (4 ROD and 3 normal) of adult female patients within ages of 67 ± 8. The control (normal bone) group samples were acquired from postmenopausal healthy women. Confirmation of ROD was also validated by histomorphometric evaluations for the other group of samples. To protect patient confidentiality, the samples were blinded by keyed numerical identification prior to shipment for current analysis. They were also standardly embedded in polymethyl methacrylate (PMMA), to facilitate cutting of 5 µm thick sections with a Leica RM 2265 microtome (Leica Biosystems Inc., Illinois, USA). A standard protocol of sample preparation for histomorphometric analysis was used, without staining.

### 2.2. Raman Measurements and Equipment

Confocal Raman microscopy was performed with an *alpha 300RAS WITec* confocal Raman system (WITec GmbH, Ulm, Germany), using a 532 nm excitation of a frequency-doubled neodymium-doped yttrium-aluminum-garnet (Nd:YAG) laser that was operated at a low power output of about 5 mW to avoid sample damage. The Raman signal was recorded with a 1024 × 127 pixel Peltier cooled back-illuminated and VIS AR–coated Marconi 40–11 charge-coupled device (CCD) with a spectral resolution of 4 wavenumbers. To particularly measure just the trabecular bone and avoid PMMA interference, specific regions of interest were carefully selected using a 20× objective lens with a 0.4 numerical aperture (Olympus, Tokyo, Japan). A low numerical aperture objective was used primarily to avoid the influence of polarization effects for phosphate and collagen amide I bands, besides to provide a greater working distance adequate for sample roughness. The *WITec Control 1.60* software was employed for confocal mapping data acquisition and for controlling the piezoelectric stage during laser scanning. Arrays of 150 × 150 Raman spectra were recorded for all Raman images using an integration time of 50 ms per spectrum. The Raman mapping images were acquired with 80 µm × 80 µm scan sizes.

### 2.3. Computational Analysis

A general linear background subtraction in the region of 377 to 1720 cm^−1^ and a normalization to the laser line intensity were first applied to each spectrum; the latter was performed to account for potential fluctuation of the laser power between measurements of different samples. To increase the accuracy of current computational analysis, before calculating the integrated areas under the relevant Raman features, an additional linear background subtraction was also performed in the characteristic frequency regions, as follows: between 395 and 469 cm^−1^ for the ν_2_PO_4_^3^ band centered at 430 cm^−1^; between 907 and 990 cm^−1^ for the ν_1_PO_4_^3^ band centered at 960 cm^−1^; between 1033 and 1135 cm^−1^ for the carbonate ν_1_CO_3_^2−^ band centered at 1074 cm^−1^; between 1215 and 1332 cm^−1^ for the amide III band centered at 1275 cm^−1^; between 1625 and 1725 cm^−1^ for the amide I band centered at 1660 cm^−1^; and between 970 and 1040 cm^−1^ and between 1574 and 1543 cm^−1^ for the two phenylalanine bands centered at 1005 cm^−1^ and 1609 cm^−1^, respectively. The ratios corresponding to the significant biomarkers, namely the mineral-to-matrix content (ν_1_PO_4_^3^/amide I ratio) [15,16,17,18,19,20,21,22,23,24,25,26,27,28,29,30], the carbonate-to-matrix (ν_1_CO_3_^2−^/amide I ratio) [15,16,17,18,19,20,21,22,23,24,25,26,27,28,29,30], the calcium content (ν_2_PO_4_^3^/amide III ratio) [31], and the phenylalanine content (phenylalanine/amide III ratio) were next calculated for each of the 7 bone samples (i.e., for each of the 22,500 × 7 = 157,500 spectra). A linear discriminant analysis (LDA) using a logit classification, which is a commonly employed approach in statistically supervised learning, was performed considering simultaneously all four biomarkers. Alternatively, the whole information contained in the Raman spectra was also evaluated through dimensionality reduction to the most relevant 20 variables, by using principal component analysis (PCA) followed by a linear support vector machine (LSVM) classification with a 10-fold cross validation, both implemented in MATLAB^®^ version r2016a. For each spectrum, a score was attributed based on the logit transformation. The reason of this prior dimensionality reduction was to decrease the computing time devoted to the LSVM algorithm.

## 3. Results and Discussion

**Figure 1:**
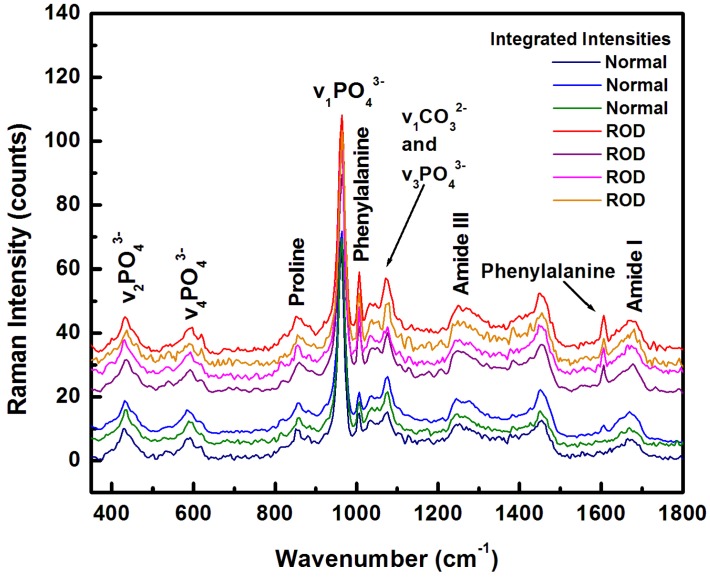
Integrated Raman spectra of 3 normal and 4 renal osteodystrophy (ROD) bone samples, each obtained by averaging 22,500 Raman spectra. The spectra are vertically translated and color labeled for easier visualization.

Since reliable ROD detection cannot be based on a single ideal biomarker, to differentiate between the normal and the ROD samples, we took advantage of the inherent Raman capability of simultaneously providing information about all significant biomarkers. In this way, we could also account for any potential changes, in a label-free and real-time manner. The integrated Raman spectra for each sample, which were obtained from averaging over 22,500 individual Raman spectra recorded per image, are presented in Figure 1.

**Figure 2:**
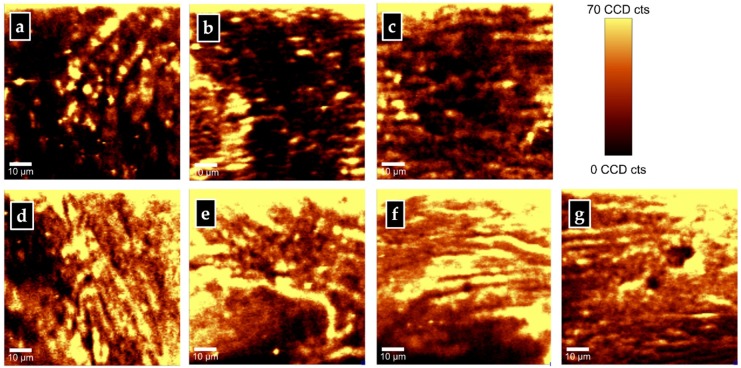
Representative confocal Raman images of phenylalanine content in: (**a**–**c**) normal bone samples and (**d**–**g**) ROD samples. A bright yellow pseudo-color corresponds to a higher Raman intensity.

Although intensity differences in some of the Raman features can be observed in these spectra, particularly for those corresponding to the phenylalanine peaks at 1005 and 1609 cm^−1^, no other evident information concerning additional biomarkers of interests can be accurately extracted without appropriate computational analysis. Indeed, the large amount of collected Raman data, besides facilitating an excellent statistics on the results (i.e., an accuracy of less than 10^−300^ for each biomarker [25]), also allows for a direct visualization by use of pseudo-color contrast of different components and their distributions. Supporting evidence are the Raman images associated with the phenylalanine content that are presented in Figure 2a–g. Generation of these images was performed by applying filters to select certain parts of the spectra, namely the frequency region from 970 to 1040 cm^−1^ for the phenylalanine peak at 1005 cm^−1^, and that from 1574 to 1543 cm^−1^ for the phenylalanine peak at 1609 cm^−1^. A brighter yellow pseudo-color in these images corresponds to a higher phenylalanine content, as the associated color scale bar reveals. An overall much larger amount of phenylalanine (more dominant yellow regions) can be observed for the ROD samples (Figure 2d–g) than for the normal bone samples (Figure 2a–c), in agreement with our previously quick examination of the spectra shown in Figure 1.

However, for in vivo data acquisition through an optical fiber-based biosensor, Raman spectral recording versus confocal Raman mapping is more suitable. Consequently, the fundamental question about the minimum number of spectra necessary to obtain a sample assessment with a sufficient (desired) accuracy still remains. We already demonstrated that by considering a single biomarker in the distinction between the samples at a typical level of significance of *p* = 0.05, about 18 spectra were needed for the mineral-to-matrix content, 20 spectra for the carbonate-to-matrix content, and 46 spectra for the calcium content [25]. The rationale implies that if we take into account in the current multivariate computational analysis all the biomarkers concurrently, a smaller number of spectra will be necessary, thus, emphasizing the possibility of ROD detection in real-time through Raman spectroscopy. It is known that the larger the number of employed variables, the more likely is to obtain a good classification power. It should also be noted here that by currently using only four variables classification (i.e., just four biomarkers), we reduce the potential impact of multicomparison correction analysis on the final *p* value [32].

**Figure 3:**
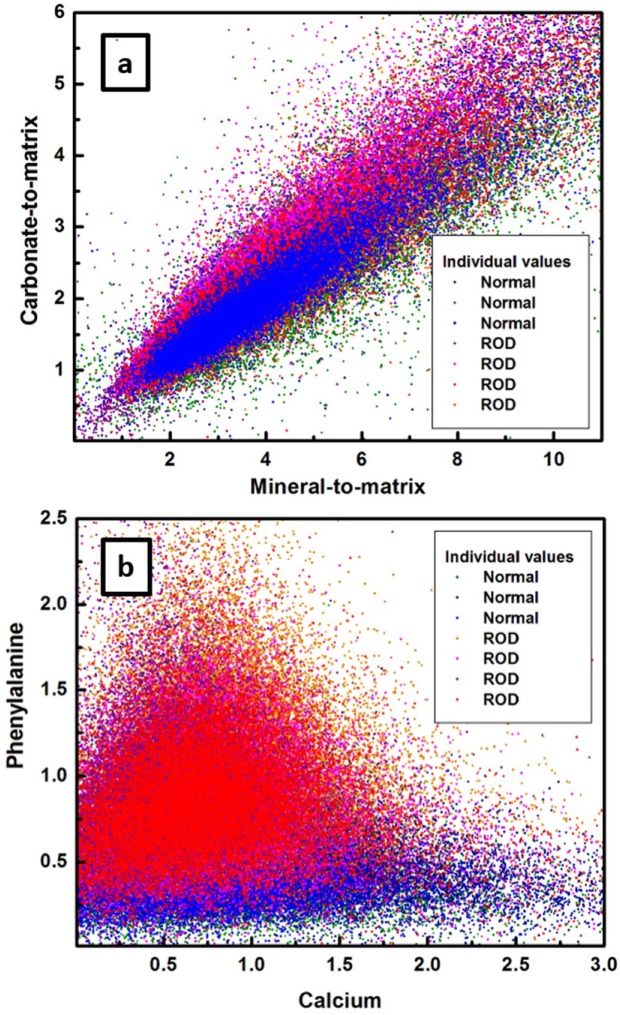
Representation of (**a**) the carbonate-to-matrix biomarker (ν_1_CO_3_^2−^/amide I) versus the mineral-to-matrix biomarker (ν_1_PO_4_^3^/amide I), and (**b**) the phenylalanine content (phenylalanine /amide III) versus that of calcium (ν_2_PO_4_^3^/amide III) for all the 22,500 independent Raman spectra measured per sample. A similar color labeling as in Figure 1 was used for each of the 7 bone samples.

Prior to finding this minimum number of spectra, a potential discrimination between the normal and ROD bone samples is attempted in Figure 3a,b through plotting of the carbonate-to-matrix component (ν_1_CO_3_^2−^/amide I ratio) versus the mineral-to-matrix component (ν_1_PO_4_^3^/amide I), and the phenylalanine content (phenylalanine/amide III) versus that of calcium (ν_2_PO_4_^3^/amide III), respectively, for each of the 22,500 Raman spectra. For consistency and because of differences in polarization sensitivities between amide I and amide III features, the mineral and carbonate contents were normalized to the amide I band, and the calcium and phenylalanine contents to the amide III band. Furthermore, to minimize the calculation errors, we consider the ratio of areas under the corresponding peaks instead of the ratio of their intensities [15,16,17,18,19,20,21,22,23,24,25,26,27,28,29,30,31]. While from Figure 3a a relatively good correlation can be implied between the mineral-to-matrix and the carbonate-to-matrix biomarkers (both being indicators of bone turnover and remodeling activity), unfortunately, only a very small differentiation between the samples can be achieved. The main reason behind this lack of sample separation is the strong overlapping between majority of the color-coded points representing independent Raman spectra. A better sample classification can be performed by examining the relationship between phenylalanine and calcium contents (see Figure 3b). The much larger amount of phenylalanine in comparison to that of calcium observed in this figure for the ROD samples (remark based on the location of these data points regarding an imaginary line of slope 1) corroborates with the clinical reports for patients with kidney malfunction (identified as ROD patients). For example, such patients demonstrate a substantially lower level of calcium in their blood test analyses [33,34,35].

**Figure 4:**
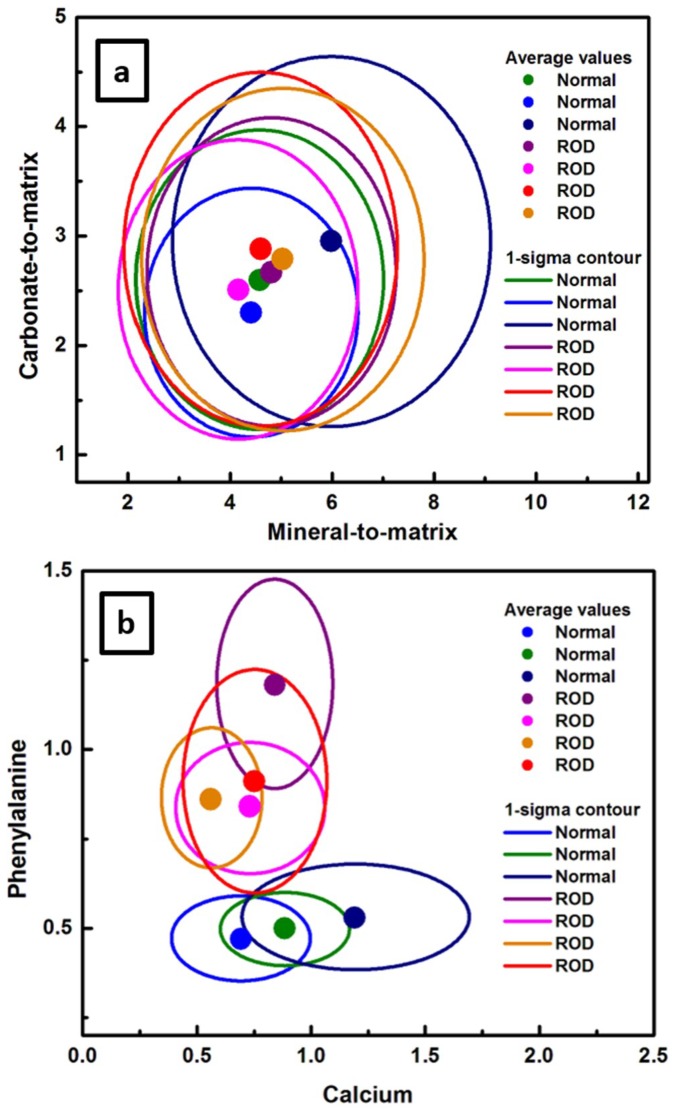
Statistical representation using 1-sigma ellipsoids of: (**a**) the carbonate-to-matrix biomarker versus the mineral-to-matrix biomarker, and (**b**) the phenylalanine content versus that of calcium. The solid circle defines the average over 22,500 spectra for each biomarker. For consistency, an identical color-code was again used.

A more compact and easier visualization of the results presented in both Figure 1 and Figure 3a,b can be obtained by plotting in Figure 4a,b the combination of these four biomarkers using statistical 1-sigma ellipsoid representations, with the biomarker averages over 22,500 spectra defined by solid circles. For consistency with Figure 3, the carbonate-to-matrix versus mineral-to-matrix is presented in Figure 4a, and phenylalanine versus calcium in Figure 4b. Another reason for using this statistical representation is to inspect for potential differences between same types of samples. Indeed, a variation from sample to sample is observed in the relationships between biomarkers, even among normal samples or ROD samples themselves. We suggest that this anticipated variation is based on age or on other specific patient conditions. However, besides a much clear distinction of phenylalanine to calcium relationship seen in Figure 4b than that observed previously in Figure 3b, no additional information regarding sample classification is attainable, even with this more compact statistical representation.

**Figure 5:**
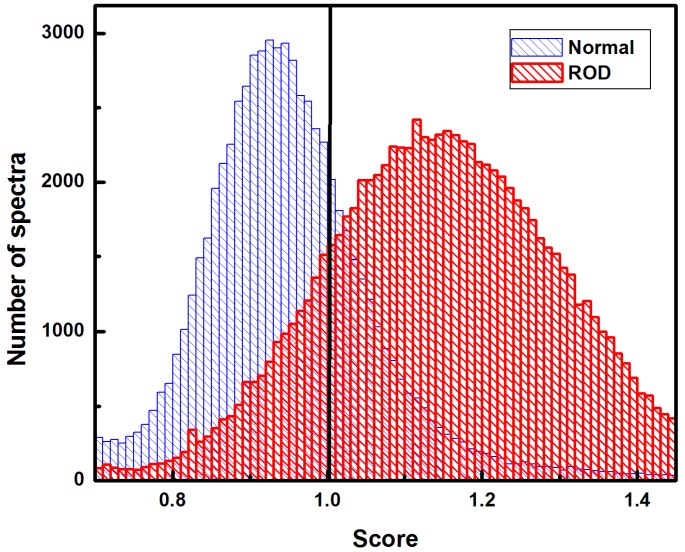
Combined histograms resulted from statistical investigations using all four biomarkers concurrently. Distribution of scores of more or less than 1 were assigned to each ROD and normal spectrum, respectively.

Therefore, we present, in Figure 5, the histograms associated with all of the above investigations (i.e., from both, Figure 3 and Figure 4), and taking as variables all the four ratios concurrently. A linear discriminant analysis with 10-fold cross validation of the training data was employed. For the prediction classification, a logistic score transformation was used, with a score less than one for normal bone spectra and more than one for ROD spectra.

The strong overlapping seen in Figure 5 between these histograms, not only agrees with the findings previously discussed, but also confirms that a sample classification cannot be based just on a single spectrum, since Type I and Type II errors will be unacceptable large in this case (see solid line at 1). A summary of the results associated with the confusion matrix and the usual parameters related to the prediction ability, which was based on randomly selecting the spectra, is presented in Table 1.

The important question is whether focusing on only four biomarkers (as measured variables) significantly affects the discrimination power of the method, since the Raman spectra in the frequency range of interest contain over 300 data points, thus, potentially over 300 independent variables. Consequently, we employed an alternative computational approach based on a linear support vector machine (LSVM) algorithm, which takes into account all of these independent variables in the classification of any unknown sample. The results associated with the confusion matrix from the LSVM are summarized in Table 2. Even though the LSVM method involves about two orders of magnitude more independent variables than does the LDA, only a marginal improvement in sample classification is accomplished based on a single spectrum. This observation, which arises from a comparison between the results presented in Table 1 and Table 2, also demonstrates that the four previously chosen variables (biomarkers selected mainly from clinical reasons) contain most of the information (discrimination power) necessary to differentiate between the normal and the ROD samples.

To improve the accuracy of the classification, we next consider N measurements of independent spectra from different locations in the sample. For N such spectra (with N being an odd integer), we assume that the sample belongs to ROD if *n* > N/2 spectra have a score greater than one. On the other hand, if *n* spectra have a score less than one, the sample is assessed as normal. Given a probability *p*_1_ that a normal spectrum has a score less than 1, and a probability *p_2_* that a ROD spectrum has a score larger than 1 (see Table 1), the Q_I_ (N) probabilities for Type I (rejection of a true null hypothesis, or false positive), and the Q_II_ (N) probabilities for Type II error (non-rejection of a false null hypothesis, or false negative) can be calculated as follows:

(1) $$Q_{I}\left(N\right)=1-P_{1}\left(N\right)=\overset{k<\frac{N}{2}}{\underset{k=0}{\sum }}\left(\begin{array}{c} N\\ k \end{array}\right){\left(1-p_{1}\right)}^{N-k}p_{1}{}^{k}$$

(2) $$Q_{II}\left(N\right)=1-P_{2}\left(N\right)=\overset{k<\frac{N}{2}}{\underset{k=0}{\sum }}\left(\begin{array}{c} N\\ k \end{array}\right){\left(1-p_{2}\right)}^{N-k}p_{2}{}^{k}$$

**Figure 6:**
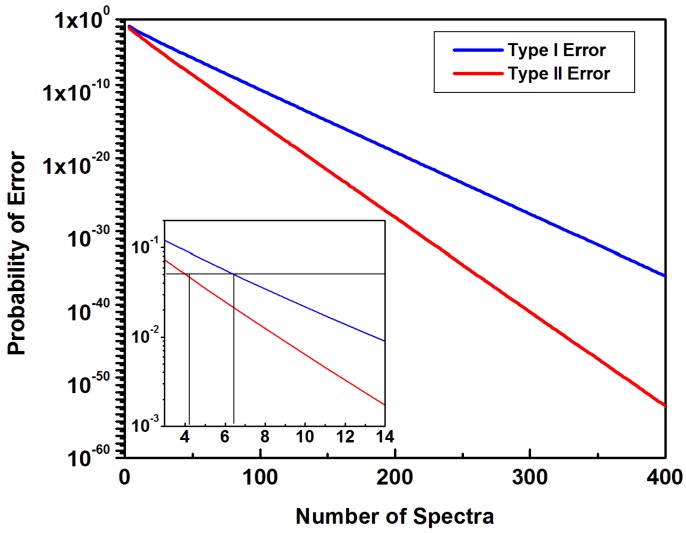
Probability of Type I and Type II errors versus the number of randomly chosen spectra employed in the classification. The black lines in the inset indicate that a relatively small set of measured spectra is sufficient to classify the samples with a typical *p* < 0.05 error probability.

The probabilities of Type 1 and Type II assignment errors, namely wrongfully assigned k = N, N−1, …, k < N/2 spectra obtained from either a normal or a ROD bone sample, are plotted in Figure 6 as a function of the number of independently recorded spectra.

While an examination of the large part of Figure 6 reveals that the assignment error probability can be made as small as desired, the inset of this figure further indicates that for a defined precision, only a low number of sampling points is necessary. For example, the black lines in the inset show that to achieve a probability of less than 5%, 7 independent spectra are sufficient for Type I error and 5 spectra for Type II error. The corresponding confusion matrix and related probabilities for 11 independent spectra are summarized in Table 3. A classification accuracy of ~99% is obtained. Since such a relatively small number of independent spectra can be in principle acquired through an optical-fiber-based biosensor (e.g., using depth profiles confocal Raman), the present work not only validate the feasibility of future in vivo Raman translation, but also emphasize the need of computational analysis for these essential predictions.

## 4. Conclusions

Ideally, ROD should be reliably detected in real time and with noninvasive or minimally invasive methods, together with the fact that its detection cannot be based on a single biomarker. This research describes alternative techniques that could provide similar or better degrees of diagnosis. It is also a logical continuation of our previous efforts in demonstrating that Raman spectroscopy can be a viable approach [25]. One of the shortcoming of the Raman technique towards its potentially clinical translation is knowing the minimum number of independent spectra that will provide an accurate assessment of this complex diseases. Another drawback is the need for development of an optical-fiber probe biosensor. To overcome the first constraint, we took advantage of both, Raman providing simultaneously information about all the biomarkers of interests in a label-free manner, and computational analysis in answering the essential question of the minimal number of spectra necessary for sample classification with a desired accuracy.

The resulting confusion matrix from a classification performed by standard LDA with 10-fold cross validation demonstrates that the probability of a correct sample assignment based on a single spectrum is about 80% (see Table 1). For this classification, a logit transform score was used for each spectrum. Moreover, the current statistical analysis shows that a reasonably small number of randomly selected spectra suffices for assessment of any sample at any desired degree of accuracy; only 7 independent spectra are necessary for Type I error and 5 spectra for Type II error. This outcome is achievable because of simultaneous consideration of all the known physical differences between the normal and ROD samples. Furthermore, the classification of bone quality on just four variables (biomarkers) reduces the potential impact of multicomparison correction analysis on the final *p* value [32]. Finally, all the information contained in the spectra was used in an alternative statistical learning algorithm for sample classification. Prior implementation of this LSVM algorithm, a dimensionality reduction by PCA in 20 directions (of most variations) was employed. Although this later classification takes into consideration much more information (~300 independent variables), the results were only marginally superior to those obtained from the LDA approach. A correct sample assignment based on a single spectrum is about 87% in this case (see Table 2).

In conclusion, the current computational study validates that only a relatively low number of spectra is necessary for accurate ROD detection, supporting the feasibility of future in vivo Raman translation through development of a biosensor for signal recording and multiplexing. This work adds value to a potentially alternative method for fast ROD assessment and human health monitoring.

## Figures and Tables

**Table 1:** Confusion matrix for single spectrum LDA classification (4 variables).

	Condition Positive	Condition Negative	Prevalence 57.14%	Accuracy 80.5%
Prediction positive	70470	11205	Precision 78.3%	FDR (false discovery rate) 16.6%
Prediction negative	19530	56295	FOR (false omission rate) 21.7%	NPV (negative predictive value) 78.3%
	Sensitivity 78.3%	Specificity 83.7%	FPR (false positive rate) 16.6%	FNR (false negative rate) 38.9%

**Table 2:** Confusion matrix for single spectrum LSVM classification (~300 variables).

	Condition Positive	Condition Negative	Prevalence 57.1%	Accuracy 87.5%
Prediction positive	70470	9112	Precision 88.3%	FDR (false discovery rate) 13.5%
Prediction negative	10530	58388	FOR (false omission rate) 11.7%	NPV (negative predictive value) 88.3%
	Sensitivity 88.3%	Specificity 86.5%	FPR (false positive rate) 13.5%	FNR (false negative rate) 15.6%

**Table 3:** Confusion matrix for 11 spectra classification.

	Condition Positive	Condition Negative	Prevalence 57.1%	Accuracy 98.8%
Prediction positive	98.3%	0.5%	Precision 98.3%	FDR (false discovery rate) 0.5%
Prediction negative	1.7%	99.5%	FOR (false omission rate) 0.0174%	NPV (negative predictive value) 98.3%
	Sensitivity 78.3%	Specificity 99.5%	FPR (false positive rate) 0.5%	FNR (false negative rate) 2.3%

## References

[1] Baldock P.A., Allison S.J., Herzog H., Gardiner E.M., Seibel M., Robins S., Bilezikian J. (2006). The Central Control of Bone Remodeling. Dynamics of Bone and Cartilage Metabolism.

[2] Shetty S., Kapoor N., Bondu J.D., Thomas N., Paul T.V. (2016). Bone turnover markers: Emerging tool in the management of osteoporosis. Indian J. Endocr. Metab..

[3] Civitelli R., Armamento-Villareal R., Napoli N. (2009). Bone turnover markers: Understanding their value in clinical trials and clinical practice. Osteoporos Int..

[4] Moe S., Drüeke T., Cunningham J., Goodman W., Martin K., Olgaard K., Ott S., Sprague S., Lameire N., Eknoyan G. (2006). Definition, evaluation, and classification of renal osteodystrophy: A position statement from Kidney Disease: Improving Global Outcomes (KDIGO). Kidney Int..

[5] Malluche H.H., Faugere M.-C. (1986). Atlas of Mineralized Bone Histology.

[6] Malluche H.H., Porter D.S., Monier-Faugere M.C., Maward H., Pienkowski D. (2012). Differences in Bone Quality in Low- and High-Turnover Renal Osteodystrophy. J. Am. Soc. Nephrol..

[7] Moorthi R.N., Moe S.M. (2013). Recent advances in the noninvasive diagnosis of renal osteodystrophy. Kidney Int..

[8] Malluche H.H., Mawad H.W., Monier-Faugere M.C. (2011). Renal osteodystrophy in the first decade of the new millennium: Analysis of 630 bone biopsies in black and white patients. J. Bone Miner. Res..

[9] Miller P.D. (2008). The role of bone biopsy in patients with chronic renal failure. Clin. J. Am. Soc. Nephrol..

[10] Morii H., Okamoto T., Iba K., Inoue T., Matsushita Y., Hasegawa K., Kikkawa T., Kanao K., Yamada N., Okamoto S. (1979). Age-related changes of renal osteodystrophy. Endocrinol Jpn..

[11] Boyce T.M., Bloebaum R.D. (1993). Cortical aging differences and fracture implications for the human femoral neck. Bone.

[12] Boskey A.L., Coleman R. (2010). Aging and bone. J. Dent. Res..

[13] Hind K., Oldroyd B., Truscott J.G. (2010). In vivo precision of the GE Lunar iDXA densitometer for the measurement of total-body, lumbar spine, and femoral bone mineral density in adults. J. Clin. Densitom..

[14] Nickolas T.L., Stein E., Cohen A., Thomas V., Staron R.B., McMahon D.J., Leonard M.B., Shane E. (2010). Bone Mass and Microarchitecture in CKD Patients with Fracture. J. Am. Soc. Nephrol..

[15] Morris M.D., Mandair G.S. (2011). Raman Assesment of Bone Quality. Clin. Orthop. Relat. Res..

[16] McNerny E.M., Gong B., Morris M.D., Kohn D.H. (2015). Bone fracture toughness and strength correlate with collagen cross-link maturity in a dose-controlled lathyrism mouse model. J. Bone Miner. Res..

[17] Esmonde-White K.A., Esmonde-White F.W., Holmes C.M., Morris M.D., Roessler B.J. (2013). Alterations to bone mineral composition as an early indication of osteomyelitis in the diabetic foot. Diabetes Care.

[18] Felice P.A., Gong B., Ahsan S., Deshpande S.S., Nelson N.S., Donneys A., Tchanque-Fossuo C., Morris M.D., Buchman S.R. (2015). Raman spectroscopy delineates radiation-induced injury and partial rescue by amifostine in bone: A murine mandibular model. J. Bone Miner. Metab..

[19] McCreadie B.R., Morris M.D., Chen T.-c., Rao D.S., Finney W.F., Widjaja E., Goldstein S.A. (2006). Bone tissue compositional differences in women with and without osteoporotic fracture. Bone.

[20] Inzana J.A., Maher J.R., Takahata M., Schwarz E.M., Berger A.J., Awad H.A. (2013). Bone fragility beyond strength and mineral density: Raman spectroscopy predicts femoral fracture toughness in a murine model of rheumatoid arthritis. J. Biomech..

[21] Burke M.V., Atkins A., Akens M., Willett T.L., Whyne C.M. (2016). Osteolytic and mixed cancer metastasis modulates collagen and mineral parameters within rat vertebral bone matrix. J. Orthop. Res..

[22] Bi X., Patil C.A., Lynch C.C., Pharr G.M., Mahadevan-Jansen A., Nyman J.S. (2011). Raman and mechanical properties correlate at whole bone- and tissue-levels in a genetic mouse model. J. Biomech..

[23] Unal M., Jung H., Akkus O. (2016). Novel Raman Spectroscopic Biomarkers Indicate That Postyield Damage Denatures Bone’s Collagen. J. Bone Miner. Res..

[24] Ding H., Nyman J.S., Sterling J.A., Perrien D.S., Mahadevan-Jansen A., Bi X. (2014). Development of Raman spectral markers to assess metastatic bone in breast cancer. J. Biomed. Opt..

[25] Ciubuc J.D., Manciu M., Maran A., Yaszemski M.J., Sundin E.M., Bennet K.E., Manciu F.S. (2018). Raman Spectroscopic and Microscopic Analysis for Monitoring Renal Osteodystrophy Signatures. Biosensors.

[26] Gourion-Arsiquaud S., Faibish D., Myers E., Spevak L., Compston J., Hodsman A., Shane E., Recker R.R., Boskey E.R., Boskey A.L. (2009). Use of FTIR spectroscopic imaging to identify parameters associated with fragility fracture. J. Bone Miner. Res..

[27] Boskey A., Mendelsohn R. (2005). Infrared analysis of bone in health and disease. J. Biomed. Opt..

[28] Boskey A., Pleshko Camacho N. (2007). FT-IR Imaging of Native and Tissue-Engineered Bone and Cartilage. Biomaterials.

[29] Paschalis E.P., Betts F., DiCarlo E., Mendelsohn R., Boskey A.L. (1997). FTIR Microspectroscopic Analysis of Human Iliac Crest Biopsies from Untreated Osteoporotic Bone. Calcif. Tissue Int..

[30] Isaksson H., Turunen M.J., Rieppo L., Saarakkala S., Tamminen I.S., Rieppo J., Kröger H., Jurvelin J.S. (2010). Infrared spectroscopy indicates altered bone turnover and remodeling activity in renal osteodystrophy. J. Bone Miner. Res..

[31] Roschger A., Gamsjaeger S., Hofstetter B., Masic A., Blouin S., Messmer P., Berzlanovich A., Paschalis E.P., Roschger P., Klaushofer K. (2014). Relationship between the V2PO4/amide III ratio assessed by Raman spectroscopy and the calcium content measured by quantitative backscattered electron microscopy in healthy human osteonal bone. J. Biomed. Opt..

[32] Wasserman L. (2003). Springer Texts in Statistics. All of Statistics. A Concise Course in Statistical Inference.

[33] Fournier A., Oprisiu R., Hottelart C., Yverneau P.H., Ghazali A., Atik A., Hedri H., Said S., Sechet A., Rasolombololona M. (1998). Renal Osteodystrophy in Dialysis Patients: Diagnosis and Treatment. Artif. Organs.

[34] Tomasello S. (2008). Secondary Hyperparathyroidism and Chronic Kidney Disease. Diabetes Spectr..

[35] Hill Gallant K.M., Spiegel D.M. (2017). Calcium Balance in Chronic Kidney Disease. Curr. Osteoporos. Rep..

